# Metabolic Effects of JAK1/2 Inhibition in Patients with Myeloproliferative Neoplasms

**DOI:** 10.1038/s41598-019-53056-x

**Published:** 2019-11-12

**Authors:** Manali Sapre, Douglas Tremblay, Eric Wilck, Annie James, Amanda Leiter, Alexander Coltoff, Anita G. Koshy, Marina Kremyanskaya, Ronald Hoffman, John O. Mascarenhas, Emily J. Gallagher

**Affiliations:** 1 Icahn School of Medicine at Mount Sinai, One Gustave L. Levy Place, New York, NY 10029 USA; 2 Division of Hematology and Medical Oncology, Icahn School of Medicine at Mount Sinai, One Gustave L. Levy Place, New York, NY 10029 USA; 3 Department of Radiology, Icahn School of Medicine at Mount Sinai, One Gustave L. Levy Place, New York, NY 10029 USA; 4 Division of Endocrinology, Diabetes and Bone Disease, Icahn School of Medicine at Mount Sinai, One Gustave L. Levy Place, New York, NY 10029 USA; 5 Department of Medicine, Icahn School of Medicine at Mount Sinai, One Gustave L. Levy Place, New York, NY 10029 USA; 6 Tisch Cancer Institute at Mount Sinai, Icahn School of Medicine at Mount Sinai, One Gustave L. Levy Place, New York, NY 10029 USA

**Keywords:** Targeted therapies, Targeted therapies

## Abstract

Ruxolitinib is an FDA approved janus kinase (JAK)1/2 inhibitor used to treat myeloproliferative neoplasms (MPNs), including myelofibrosis and polycythemia vera. We aimed to determine the metabolic consequences of ruxolitinib treatment in patients with MPNs. We performed a retrospective single-center cohort study utilizing an electronic medical record based database of patients who began treatment with ruxolitinib for MPNs from January 2010 to March 2017. We also examined the effects of ruxolitinib on adipose tissue JAK/STAT signaling in a mouse model. 127 patients were identified, of which 69 had data available for weight, and at least one other parameter of interest before, and 72 weeks after starting ruxolitinib. Mean baseline weight was 73.9 ± 17.0 kg, and 78.54 ± 19.1 kg at 72 weeks (p < 0.001). 50% of patients gained >5% body weight. Baseline body mass index (BMI) was 25.8 ± 4.8 kg/m^2^, and 27.5 ± 5.5 kg/m^2^ at 72 weeks (p < 0.001). Patients treated with ruxolitinib had a higher systolic blood pressure, serum AST, and ALT at 72 weeks, compared with baseline (p = 0.03, p = 0.01, p = 0.04, respectively). In mice, ruxolitinib decreased basal and GH-stimulated STAT5 phosphorylation in adipose tissue. As pharmacological JAK1/2 inhibitors are being developed and used in clinical practice, it is important to understand their long-term metabolic consequences.

## Introduction

Janus kinase (JAK) phosphorylation is a key step in the activation of the intracellular signaling pathways for a number of cytokines and growth factors^1^. Dysregulated JAK/signal transducer and activator of transcription (STAT) signaling is the central pathogenic mechanism in myeloproliferative neoplasms (MPNs) which are associated with mutations in JAK2 and the thrombopoietin receptor (MPL) genes, and calreticulin. These abnormalities lead to elevated levels of circulating cytokines, in addition to autocrine and paracrine cytokine signaling^2–4^. Therefore, therapeutic inhibition of JAK/STAT signaling is routinely employed in the treatment of MPN patients^5^.

Ruxolitinib (INCB018424, Incyte, Wilmington, DE) is a small molecule JAK1/2 tyrosine kinase inhibitor that is approved by the US Food and Drug Administration (FDA) to treat certain MPNs, including myelofibrosis (MF) and polycythemia vera (PV)^6–8^. A post-hoc analysis of phase III clinical trials reported a mean weight gain of 3.9 kg in patients with MF treated with ruxolitinib at 24 weeks^9^; however, a detailed examination of the distribution of weight gain, and the metabolic implications of JAK1/2 inhibition with ruxolitinib in patients with MF and PV, has not been described.

Our current understanding of the role of JAK/STAT signaling in the regulation of metabolism has largely been derived from tissue-specific deletions of Jak2, Jak3, Stat3, and Stat5 in murine models^1^. However, the metabolic effects of systemic inhibition of Jak1 or Jak2 signaling have not been studied in animal models, as deletion of Jak1 or Jak2 leads to embryonic or neonatal death in murine models^1^. Tissue-specific knockout studies have demonstrated that JAK/STAT signaling regulates multiple metabolic factors, including adiposity, energy expenditure, glucose tolerance, insulin secretion and sensitivity, lipid regulation, hepatic steatosis, and inflammation^1^. Overall, JAK/STAT signaling confers complex effects on metabolic physiology and pathophysiology^1^.

The aim of our study was to determine the metabolic consequences of JAK1/2 inhibition with ruxolitinib in patients with MF and PV. We also aimed to determine how JAK1/2 inhibition affects adipose tissue signaling in correlative preclinical studies.

## Methods

### Patient selection

We conducted a retrospective cohort study of patients with MPNs who initiated ruxolitinib treatment from January 2010 to March 2017 and were treated at the Hematology-Oncology clinical facilities in the Icahn School of Medicine at Mount Sinai, New York, NY. Patients were identified from the hospital’s electronic medical record (EMR) system. Information was collected by medical record review and entered into the web-based data capture tool REDCap (Research Electronic Data Capture) hosted by the Icahn School of Medicine at Mount Sinai^10^. The Institutional Review Board of the Icahn School of Medicine at Mount Sinai (#17-02299) approved the study and waived the need for informed consent. All research was performed in accordance with the IRB approved protocol.

### Data acquisition

Data extracted from EMRs included: sex; age; date of birth; indication for starting ruxolitinib; date of ruxolitinib initiation and discontinuation; duration of follow up after ruxolitinib initiation; measurement of baseline and follow up body weight, height, body mass index (BMI); diastolic and systolic blood pressure (DBP and SBP); random serum glucose; complete blood count; peripheral blood smear; hemoglobin A1c (HbA1c); lipid profiles; serum alanine aminotransferase (ALT); serum aspartate aminotransferase (AST); medications to treat or a diagnosis of hypertension, a history of diabetes mellitus or dyslipidemia; abdominal CT scan images and dates, documentation of splanchnic vein thrombosis, splenomegaly and the extent of splenomegaly. Body weight, BMI, DBP, SBP, and lab values were collected at baseline (up to 1 year prior to starting ruxolitinib) and 72 ± 8 weeks after starting ruxolitinib. Medications and diagnoses were recorded from the medical records before and after the initiation of ruxolitinib treatment.

### Patient exclusions

As the purpose of our study was primarily to examine the effect of systemic JAK1/2 inhibition on body weight, patients who did not have a documented weight prior to initiating ruxolitinib and 72 weeks after initiating ruxolitinib were excluded. Additionally, individuals were excluded for having no additional metabolic information (blood pressure or lab values).

### Definitions of metabolic disease

BMI was used to define underweight, normal weight, overweight and obesity, as waist circumference was not documented in the medical records. Weight categories were defined as follows: underweight BMI <18.5 kg/m^2^; normal 18.5–24.9 kg/m^2^; overweight 25–29.9 kg/m^2^; obese ≥30 kg/m^2^. Elevated blood pressure was defined by the metabolic syndrome criteria as a SBP ≥130 mmHg; or DBP ≥85 mmHg; or documented diagnosis, or prescription for a medication to treat elevated blood pressure. Hyperglycemia was defined as a random glucose ≥200 mg/dL (fasting glucose was not available), or a documented diagnosis of diabetes, or prescription for medication to treat elevated blood glucose^11^.

### Measurement of adiposity on CT imaging

Visceral and subcutaneous adipose tissue masses were quantified on abdominal CT at the third lumbar (L3) region using ImageJ (NIH, Bethesda, MD) using the methods previously described^12^.

### Prognostic scoring system

Dynamic International Prognostic Scoring System (DIPSS) scores were calculated for the patients with MF at baseline^13^. DIPSS scores are used as a prognostic indicator of survival for patients with primary MF. Higher DIPSS scores are indicative of a worse prognosis. DIPSS scores were quantified for patients in the group with MF using patient age, white blood cell (WBC) count, hemoglobin level, and presence of circulating blasts on peripheral blood smear review at baseline, using methods previously described^13^. Constitutional symptoms (night sweats, fevers, weight loss >10%) were excluded from analysis as these are unreliably documented. Points were calculated as follows: 1 point each was given for: age >65 years, WBC > 25 × 10^9^/L, peripheral blood blasts ≥1%; 2 points were given for hemoglobin <10 g/dL. Patients were categorized into DIPSS risk groups as follows: low risk was 0 points, intermediate-1 was 1-2 points, intermediate-2 was 3–4 points, and high risk was >5 points. We also recorded the presence of splanchnic vein thrombosis, and quantified the extent of splenomegaly by palpation at baseline. Severity of splenomegaly was defined as: minimal <5 cm, moderate 5–10 cm, and massive >10 cm below the left costal margin.

### Statistical analysis

Statistical analyses were performed using SPSS Statistics 25 (IBM, Armonk, NY). Results are expressed as the mean of the group ± standard deviation, unless otherwise indicated. A Wilcoxon signed rank test was used to compare parameters between baseline and 72 weeks after initiation of ruxolitinib treatment.

### Animal studies

Animal studies were approved by the Institutional Animal Care and Use Committee (IACUC) of the Icahn School of Medicine at Mount Sinai in accordance with the Guide for the Care and Use of Laboratory Animals. C57BL/6J mice were purchased from The Jackson Laboratory (Bar Harbor, ME Stock number 000664). Mice were housed in the Icahn School of Medicine at the Mount Sinai Center for Comparative Medicine and Surgery. Non-fasted adult male mice were used for these experiments. Mice were fed with Rodent Diet 20 5053 (regular chow) or 5058 (high fat diet), as indicated. Ruxolitinib (LC Laboratories, Woburn, MA) 60 mg/kg or vehicle were administered by oral gavage 60 minutes prior to euthanasia. Recombinant human growth hormone (rhGH, Novo Nordisk, Copenhagen, Denmark) 125 ug/kg or vehicle was administered by intraperitoneal injection 15 minutes prior to euthanasia. Animals were euthanized, and gonadal adipose tissues were removed, and flash frozen in liquid nitrogen for subsequent analysis.

### Protein isolation and western blot analysis

Western blot analysis was performed as previously described^14^. Primary antibodies and dilutions used were: phosphorylated STAT5 (Tyr694) 1:1000 dilution (#9359, Cell Signaling Technology, Danvers, CA); STAT5 1:200 dilution (SC-835, Santa Cruz, CA); beta-actin 1:5000 (A1978 Sigma, St. Louis, MO). Anti-rabbit and anti-mouse secondary antibodies were from Li-cor Biosciences (Lincoln, NE). Membranes were imaged using the Li-cor imaging system.

## Results

### Patients and characteristics

**Figure 1:**
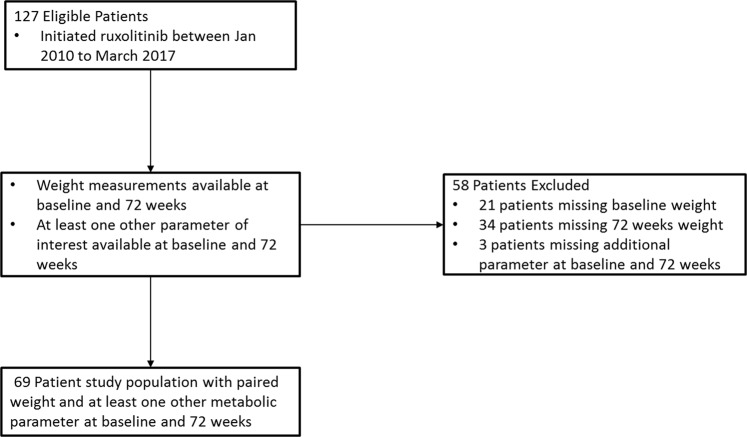
Flowchart illustrating exclusion and inclusion criteria of patients in this study.

127 patients were initially identified as being treated with ruxolitinib between January 2010 and March 2017, of which 58 were excluded due to missing data (Fig. 1). 69 patients had data available for weight, and at least one other metabolic parameter of interest at baseline and 72 weeks after initiation of treatment. 52 (75.4%) patients received ruxolitinib for the treatment of MF, 14 (20.3%) for PV; and 3 (4.3%) for other MPNs. Baseline patient characteristics for the entire group, and the subsets of patients with MF and PV are shown in Table 1.

**Table 1:** Baseline Patient Characteristics*.

Variable	All Patients (N = 69)	MF (N = 52)	PV (N = 14)	P Value
Female sex (% of patients)	35 (50.7)	28 (53.8)	5 (35.7)	P = 0.167
Age at initiation of ruxolitinib (range), years	65 (41-86)	64 (41-85)	68 (55-85)	P = 0.499
Weight, kg	73.9 (±17.0)	73.0 (±17.0)	77.8 (±19.1)	P = 0.591
BMI, kg/m2 (n = 68)	25.8 (±4.8)	25.8 (±4.8)	25.6 (±5.1)	P = 0.992
Obesity, %	10 (14.5)	9 (17.3)	1 (7.7)	P = 0.594
SBP (mmHg) (n = 68)	124 (±14.7)	124 (±13.9)	125 (±17.3)	P = 0.981
DBP (mmHg) (n = 68)	72 (±8.4)	71 (±8.7)	74 (±8.3)	P = 0.541
Hypertension, (%) (n = 68)	44 (63.8)	33 (63.5)	8 (57.1)	P = 0.594
Glucose, mg/dL (n = 55)	100 (±39.6)	101 (±41)	89 (±21)	P = 0.440
ALT, IU/L (n = 55)	25.8 (±16.0)	25.8 (±16.4)	22.7 (±9.1)	P = 0.904
AST, IU/L (n = 55)	27.0 (±11.5)	27.5 (±11.8)	21.6 (±5.9)	P = 0.337

*3 patients had an indication besides MF and PV and were categorized as “Other.” Data are mean ± SD.

In the total group, there were 34 (49.3%) males and 35 (50.7%) females. The age range was 41 to 86 years, with a mean age of 65 years. The mean baseline BMI was 25.8 kg/m^2^ and ranged from 16.8 to 41.1 kg/m^2^. Interestingly, although cachexia is thought to be a more prominent feature of MF than PV, the mean baseline BMI of patients with MF was not lower than that of patients with PV. One patient with PV was obese at baseline, while 9 (17.3%) patients with MF were obese at baseline (Table 1). We hypothesized that the higher than expected BMI in the patients with MF may be related to increased body weight due to fluid retention, or splenomegaly in those with higher DIPSS scores. Of the 52 patients in the MF group, 50 had sufficient data documented in the medical records to calculate DIPSS scores. We found that those with higher DIPSS scores did not have a higher BMI, and more than two-thirds of the group had low risk or intermediate-1 DIPSS scores (Table 2). Similarly, patients with moderate and severe splenomegaly did not have a higher BMI than those with mild splenomegaly (Table 2). Therefore, the higher than expected baseline BMI in the MF group was not influenced by DIPSS score, or spleen size.

**Table 2:** DIPSS Score and Spleen Size in MF group at Baseline*.

DIPSS	N	%	BMI (kg/m2)	Spleen Size	N	%	BMI (kg/m2)
Low risk	10	20.0%	26.4 ± 4.8	Mild	18	36.7%	28.0 ± 4.8
Intermediate-1	24	48.0%	26.1 ± 5.0	Moderate	12	24.5%	24.6 ± 5.2
Intermediate-2	14	28.0%	25.5 ± 5.0	Severe	19	38.8%	25.2 ± 5.2
High risk	2	4.0%	22.9 ± 6.0				

*2 patients did not have data available for DIPSS score. 3 patients did not have data available for spleen size. BMI data are mean ± SD.

### Effect of ruxolitinib on body weight and BMI

**Figure 2:**
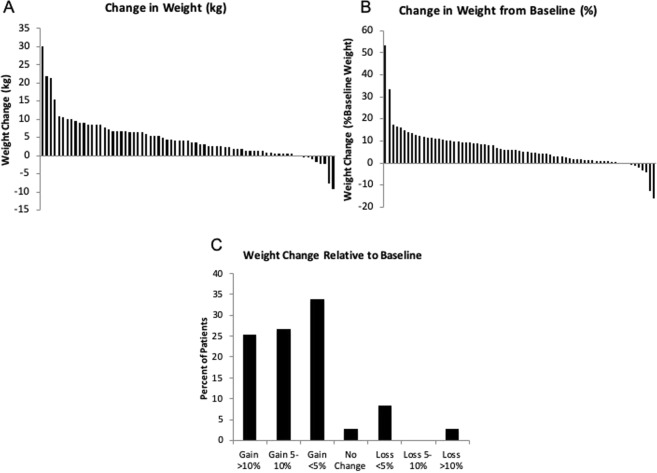
Weight changes in patients treated with ruxolitinib from baseline to 72 weeks after treatment initiation. (**A**) Change in absolute weight, (**B**) percent change in weight from baseline body weight, and (**C**) percent of patients in the cohort that gained or lost >10%, 5–10%, <5% or had no change in body weight, as indicated.

Mean weight was 73.9 ± 17.0 kg at baseline, and was 78.5 ± 19.1 kg after 72 weeks of ruxolitinib treatment (p < 0.001, Table 3). The absolute weight and percent body weight change from baseline for each individual patient are shown in Fig. 2A, B, respectively. Overall, 24.6% (n = 17) gained >10% body weight; 26.1% (n = 18) gained 5–10% body weight; 34.7% (n = 24) gained <5% body weight, and the remaining patients experienced no weight change or lost weight (Fig. 2C).

**Table 3:** Comparison of Metabolic Parameters*.

Variable	Baseline	72 weeks	P value
Weight (kg) (n = 69)	73.9 (±17.0)	78.5 (±19.1)	P < 0.001
BMI (kg/m2) (n = 68)	25.8 (±4.8)	27.5 (±5.5)	P < 0.001
Underweight (%)	2.9	1.5	P = 0.013
Normal (%)	41.2	38.2	
Overweight (%)	41.2	30.9	
Obese (%)	14.7	29.4	
Glucose (mg/dL) (n = 55)	100 (±40)	101 (±30)	P = 0.037
Hyperglycemia (%)	8 (14.5)	8 (14.5)	P = 1.000
SBP (mmHg) (n = 68)	124 (±14.7)	129 (±18.8)	P = 0.031
DBP (mmHg) (n = 68)	72 (±8.4)	70 (±7.3)	P = 0.135
Hypertension (%)	44 (64.7)	47 (69.1)	P = 0.727
ALT (IU/L) (n = 55)	25.8 (±16.0)	32.1 (±19.1)	P = 0.013
AST (IU/L) (n = 55)	27.0 (±11.5)	31.5 (±13.0)	P = 0.041

*Data are mean ± SD.

Mean BMI at baseline was 25.8 ± 4.8 kg/m^2^, and 27.5 ± 5.5 kg/m^2^ at 72 weeks (p < 0.001). At baseline, 2.9% were underweight, 41.2% normal weight, 41.2% overweight, and 14.7% obese. By 72 weeks, the proportion of obese patients almost doubled from baseline, with a distribution of 1.5% underweight, 38.2% normal weight, 30.9% overweight, and 29.4% obese. At 72 weeks, 20.6% of patients had moved up a BMI class from baseline.

### Adipose tissue distribution on abdominal CT

**Figure 3:**
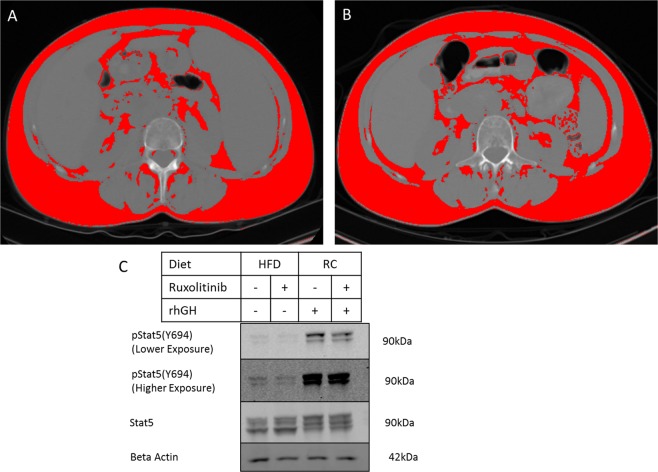
Changes in adipose tissue mass and signaling after ruxolitinib treatment. Abdominal CT images at lumbar vertebra L3 level before (**A**) and after (**B**) treatment with ruxolitinib in a patient with 4.1 kg (6.5%) body weight gain. Red coloration indicates adipose tissue. (**C**) Western blot analysis of STAT5 phosphorylation in gonadal adipose tissue from C57BL/6J mice fed high fat diet (HFD), or regular chow (RC), and acutely treated with ruxolitinib, rhGH or vehicle, as indicated.

To explore the distribution of the weight gain, we identified patients who had abdominal CT scans before starting ruxolitinib and near the 72-week timepoint. Four patients had abdominal CT scans both before and after starting ruxolitinib, however two patients had baseline CT scans more than a year prior to starting ruxolitinib, and one patient only had a follow up scan 28 weeks after starting ruxolitinib. Only one of the four patients had gained weight and had a CT scan close to ruxolitinib initiation (10 weeks prior to initiating ruxolitinib, Fig. 3A), and the 72 week time-point (88 weeks after starting ruxolitinib) (Fig. 3B). Using the methods previously described^12^, this patient was found to have an increase in waist circumference (926 mm at baseline, 966 mm at 88 weeks). The total fat area increased from 33.6% of the total abdominal area at baseline, to 41.2% at 88 weeks. There were increases in the absolute area of both visceral and subcutaneous adipose tissue depots. However, the ratio of visceral fat to subcutaneous fat increased from 16.9% visceral/83.1% subcutaneous fat at baseline, to 25% visceral/75% subcutaneous at 88 weeks. As excess abdominal adipose tissue is strongly associated with the risk of metabolic disease^15^, these changes suggested that the weight gain associated with ruxolitinib could be associated with adverse metabolic consequences.

### Effects of ruxolitinib on metabolic parameters

To determine if the weight gain associated with ruxolitinib could contribute to adverse metabolic effects on glucose homeostasis, cardiovascular risk or hepatic steatosis, we next investigated the effect of ruxolitinib treatment on blood pressure, glucose concentrations and hepatic enzymes (Table 3). In the total group, SBP increased from 124 ± 15 mmHg at baseline to 129 ± 19 mmHg at 72 weeks, (p = 0.031, n = 68). DBP was not different between baseline and 72 weeks. The percent of patients with hypertension at baseline (defined as systolic blood pressure ≥130 mmHg; or diastolic blood pressure ≥85 mmHg; or documented diagnosis of hypertension, or prescription for a medication to treat elevated blood pressure) was 64.7% and at 72 weeks was 69.1%. Glucose at baseline was 100 ± 40 mg/dL and 101 ± 30 mg/dL at 72 weeks (p = 0.037, n = 55). There was no change in the percent of patients with hyperglycemia and/or diabetes at baseline (14.5%) and 72 weeks (14.5%). Increases in both serum ALT (25.8 ± 16.0 IU/L vs 32.1 ± 19.1 IU/L, p = 0.013) and AST (27.0 ± 11.5 IU/L vs 31.5 ± 13.0 IU/L, p = 0.041) were noted at 72 weeks.

### Acute *in vivo* effects of ruxolitinib on adipose tissue GH signaling

As JAK/STAT signaling has previously been reported to be an important regulator of adipose tissue lipolysis, and GH signaling in adipose tissue has been found to activate lipolytic signaling^16^, we hypothesized that JAK1/2 inhibition may contribute to increased accumulation of adipose tissue by directly inhibiting JAK/STAT signaling in adipose tissue. Acute treatment with ruxolitinib in C57BL/6J mice fed a high fat diet reduced basal levels of STAT5 phosphorylation in adipose tissue. (Fig. 3C). Additionally, we found that ruxolitinib treatment attenuated the effect of rhGH on STAT5 phosphorylation (Fig. 3C). These results suggest that ruxolitinib may in part induce weight gain through inhibition of adipose tissue JAK/STAT signaling.

## Discussion

This study shows that in clinical practice, pharmacological JAK1/2 inhibition with ruxolitinib led to clinically significant weight gain of more than 5% baseline body weight in over half of patients treated. Additionally, ruxolitinib treatment was associated with increased systolic blood pressure, and a small rise in the random glucose, ALT and AST concentrations. Our exploratory investigation of the CT imaging from one patient that gained weight over the 72 week treatment period suggested that ruxolitinib leads to weight gain by promoting adipose tissue accumulation. Furthermore, our studies in a murine model suggested that this accumulation in adiposity may be related to direct inhibition of JAK/STAT signaling in adipose tissue.

Clinical trials have reported that ruxolitinib contributes to a mean weight gain in patients with MF^7^, however our study shows that a number of patients gain substantially more weight than previously reported. One proposed mechanism of weight gain in patients treated with ruxolitinib is through a reduction in inflammatory cytokines, which is associated with decreased spleen size and improved total symptom scores^7^. However, a post-hoc analysis of the COMFORT-I study found that ruxolitinib treatment led to weight gain regardless of the degree of reduction in spleen size, or total symptom score^9^, suggesting that patient weight gain was not dependent on the resolution of the ongoing inflammation. Given that increased inflammatory response is a driver of cardiac and metabolic complications in obese patients^17^, it would be interesting to investigate any correlation between weight gain and levels of CRP or other pro-inflammatory cytokines in patients treated with ruxolitinib in a future study. We had limited information in this study on the changes in body composition, due to minimal imaging data. We found an increase in the ratio of visceral to subcutaneous fat; however, the apparent increase in visceral adiposity may be related to the reduction in spleen size. In future studies measurements of waist circumference could be performed which may help to determine the extent to which the increase in BMI correlates with increase in abdominal obesity^18^. However, patients with MF and a higher DIPSS score may have a greater degree of splenomegaly, fluid retention and ascites, which would also increase the waist circumference. Based on previously published pre-clinical studies on adipocyte-specific knockout of JAK2, pharmacological inhibition of JAK2 would be anticipated to impair lipolysis, normally mediated by growth hormone and leptin signaling, leading to weight gain^1^. Indeed, in our study we find that ruxolitinib did impair basal and GH stimulated signaling in rodent adipose tissue, suggesting that ruxolitinib may have direct effects on adipose tissue metabolism in patients that gain weight. At present it is unclear why some patients gain substantially more weight than others, but the differences in weight gain in response to JAK1/2 inhibition may be partially explained by polymorphisms in JAK2 or in the STAT5B binding region of the peroxisome proliferator-activated receptor gamma gene promoter^19–21^. Our study lacked sufficient molecular data to explore a correlation between driver mutation status and JAK inhibitor associated weight gain.

The elevation in hepatic transaminases that we observed in these patients may be an indicator of hepatic steatosis. However, we cannot rule out the possibility that ruxolitinib may lead to drug induced liver injury in some cases. The preclinical models of hepatic Jak2 gene deletion reported that hepatic lipid accumulation and steatosis occurred, but without worsening of insulin resistance^22,23^. In our study an insufficient number of patients had circulating lipid profiles drawn as part of their usual clinical care, making it impossible to assess the effects of ruxolitinib on circulating triglycerides. We also observed a small increase in serum glucose levels of uncertain clinical significance. There are numerous cautions in interpreting any effect of JAK1/2 inhibition on glucose homeostasis in this study, due to the fact that the serum glucose levels were not measured after fasting. An insufficient number of patients had HbA1c. HbA1c levels in patients with hematological malignancies are also frequently an unreliable indicator of glucose control because of the need for therapeutic phlebotomies and transfusions which can alter A1c levels^24^. The pre-clinical studies on Jak2 deletion have reported conflicting results regarding the effects on insulin sensitivity and glucose tolerance^1^; therefore, further prospective studies will be needed in patients to understand the effects of pharmacological JAK1/2 inhibition on glucose homeostasis.

Previous studies have not reported any changes in blood pressure in response to ruxolitinib treatment. JAK2 is known to be involved in endothelial cell, smooth muscle, and cardiac function. JAK2 deletion or pharmacological inhibition of JAK2 has been reported to impair the expression of endothelial NOS^25,26^, which potentially might explain the increase in SBP observed here. Although the increase in SBP observed in this cohort was small, previous pharmacological studies, including those examining inhibitors of the cholesteryl ester transfer protein (CETP), found that small increases in systolic blood pressure were associated with an increased frequency of cardiovascular events^27^. Patients with PV are at high risk for venous and arterial thrombotic events, which are a major cause of mortality. Recognizing and treating co-morbidities that also increase cardiovascular risk such as diabetes, dyslipidemia, and hypertension is important to reduce the overall risk of thrombosis in these patients. However, ruxolitinib is known to reduce pro-inflammatory cytokines in patients with MPNs^9^. Reducing the circulating concentrations of these pro-inflammatory cytokines may provide protection from cardiovascular risk. When evaluating our findings, it is important to take into consideration that there is a lack of research investigating long-term cardiovascular morbidity and mortality in patients treated with ruxolitinib, and our study does not include cardiovascular events or mortality outcomes. Thus, we cannot make a judgement about its long-term metabolic effects, positive or negative, which remains an important avenue for future research.

The unknown long-term consequences of ruxolitinib-associated weight gain is a challenge for the practicing physician deciding how to best manage weight gain in these patients. Considering that the majority of patients in our study (50.7%) gained >5% of baseline body weight while taking ruxolitinib, we would advise pre-treatment weight counseling for all patients prior to starting ruxolitinib, encouraging attention to diet and physical activity during treatment. We would advise screening for hypertension and abnormal liver function tests in patients being treated with ruxolitinib. If evidence of secondary metabolic effects due to weight gain are found, or if the weight gain becomes bothersome to the patient, further lifestyle interventions or pharmacological interventions could be considered. However, it is important to stress that it remains unknown if ruxolitinib-associated weight gain will be modified by diet, exercise, and pharmacologic interventions, or if treating weight gain in these patients will improve morbidity and mortality.

## Conclusion

As cancer therapies improve, and patients are living longer on such therapies, understanding the long-term consequences of these targeted therapies on metabolic health is increasingly important. Based on the results of our retrospective study, we recommend monitoring patients who are treated with JAK targeted therapy for the development of metabolic derangements including obesity, hyperglycemia, dyslipidemia, hypertension and hepatic steatosis. Further studies are needed to evaluate whether these metabolic derangements are associated with changes in cardiovascular morbidity and mortality in this group of patients.

## Supplementary information

Supplementary file
