# Supplementary material for: Metabolic Effects of JAK1/2 Inhibition in Patients with Myeloproliferative Neoplasms
Source: Sci Rep. 2019 Nov 12;9:16609. doi: 10.1038/s41598-019-53056-x (PMC6851362; doi:10.1038/s41598-019-53056-x)

Supplementary Data

Title: Metabolic Effects of JAK1/2 Inhibition in Patients with Myeloproliferative Neoplasms

Authors: Manali Sapre, Douglas Tremblay, Eric Wilck, Annie James, Amanda Leiter, Alexander Coltoff, Anita Geevarghese, Marina Kremyanskaya, Ronald Hoffman, John O. Mascarenhas, Emily J. Gallagher.

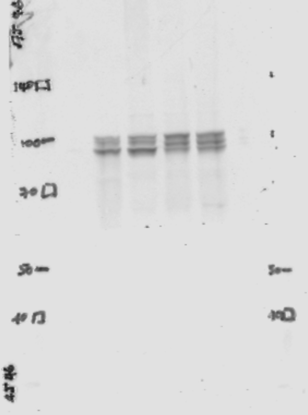
140
100
70
50
40
50
40

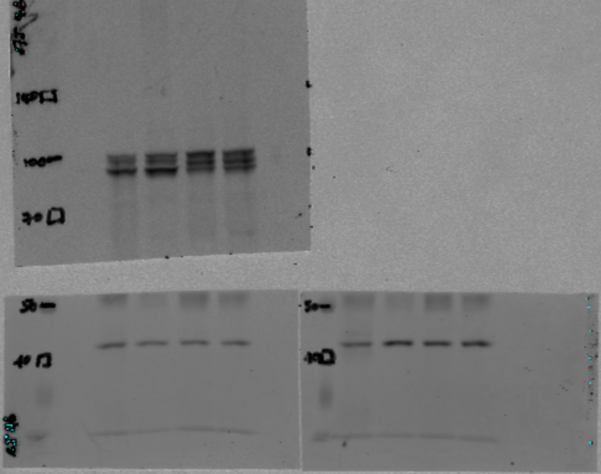
100
70
50
40

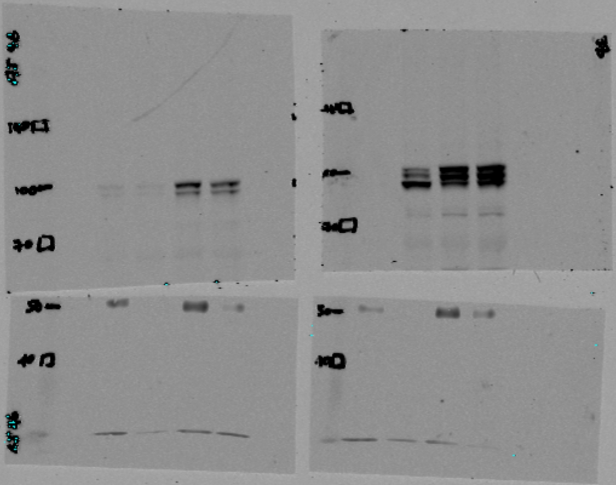

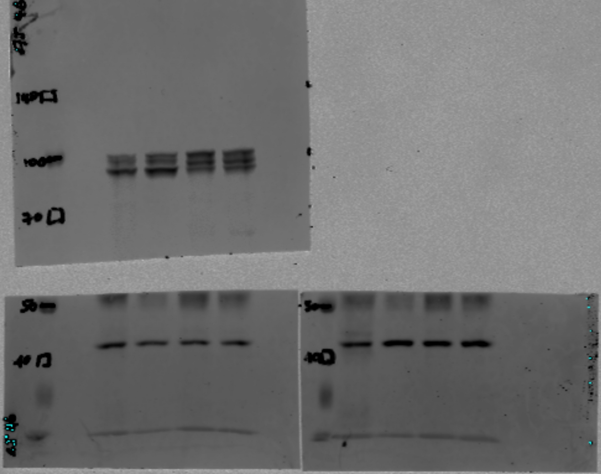
100
70
50
40
50
40

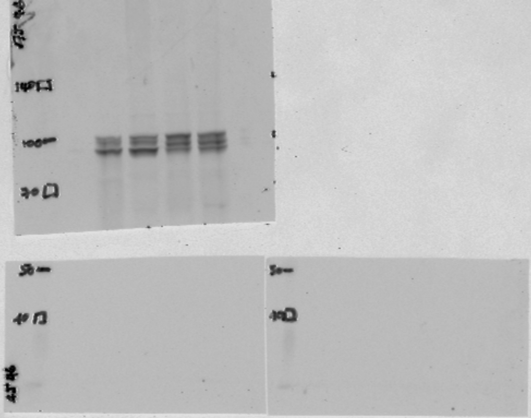
100
70
50
40
50
40

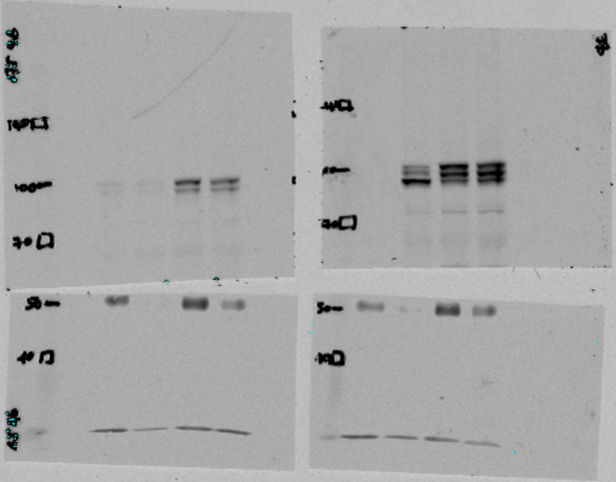

Supplement: Supplementary file 1 — Supplementary file [file 41598_2019_53056_MOESM1_ESM.pdf]
